# The Instantaneous Effect and Its Mechanism of Transcranial Direct Current Stimulation on Working Memory Based on Delta and Gamma Band Electroencephalography Characteristics

**DOI:** 10.3390/brainsci15060579

**Published:** 2025-05-27

**Authors:** Pengyi Lu, Hongli Yu

**Affiliations:** 1 State Key Laboratory of Reliability and Intelligence of Electrical Equipment, Hebei University of Technology, Tianjin 300130, China; m15688159010@163.com; 2 Tianjin Key Laboratory of Bioelectromagnetic Technology and Intelligent Health, Hebei University of Technology, Tianjin 300130, China

**Keywords:** electroencephalography, transcranial direct current stimulation, working memory, phase–amplitude coupling, power spectral density, brain network

## Abstract

Background: Working memory (WM) is a critical component of cognitive performance. Although transcranial direct current stimulation (tDCS) is a promising neuromodulation technique, its effect on working memory in healthy individuals remains unclear. Methods: In this study, EEG signals were recorded from different groups (working memory group, tDCS group, and sham group) and analyzed using phase–amplitude coupling, power spectral density, and brain network analysis to investigate the effect and mechanism of tDCS on working memory tasks in healthy individuals. Results: The results showed that in the tDCS group, the power spectral density of the EEG was increased in the gamma band and decreased in the delta band, while the delta–gamma phase–amplitude coupling was also decreased during the working memory task. Meanwhile, brain network analysis indicated significant differences in the node degree and clustering coefficient between the active and sham stimuli. Conclusions: Despite the absence of immediate behavioral improvements, these results suggest that tDCS could significantly modulate working memory-related EEG activity.

## 1. Introduction

Working memory serves as the foundation for the development of higher cognitive functions, including language comprehension, learning, and reasoning [1]. Declining quality of working memory is often associated with Alzheimer’s disease, schizophrenia, depression, and other cognitive disorders [2,3,4]. In recent years, substantial improvements in living standards and technological advancements have heightened concerns regarding working memory in healthy individuals. Consequently, it is becoming increasingly significant to find neuromodulatory methods that are capable of effectively enhancing working memory.

Transcranial direct current stimulation (tDCS) has rapidly gained traction due to its ease of operation and non-invasive characteristic, making it a prominent focus in the fields of cognitive neuroscience, neurorehabilitation, and psychiatry. However, although many studies have reported that tDCS can have an impact on working memory, the limitations and inconsistency of its effect have begun to emerge and the instantaneous effect and its mechanism of tDCS’s influence on working memory remains to be explored [5,6,7]. Some studies have demonstrated that tDCS can significantly improve working memory capacity both during and after stimulation [8,9,10,11]. Combining this non-invasive neurostimulation with cognitive training has also been shown to better improve working memory compared to cognitive training alone [12]. However, some experiments have shown no significant improvement in working memory after tDCS stimulation, regardless of whether the subjects were healthy individuals or cognitively impaired patients [13]. Some systematic reviews have shown that although some studies have shown that tDCS has a positive effect on working memory, most studies have failed to provide consistent evidence to support the significant improvement of tDCS on working memory, with large heterogeneity [14,15,16,17]. When Nejati explored the impact of tDCS on the cognitive function of patients with attention deficit and hyperactive disorder, it was found that the tDCS in the left dorsolateral prefrontal lobe of the anode could have a more significant impact on cognitive executive abilities, including working memory [18]. Jones believes that tDCS combined with cognitive training can enhance WM performance by regulating the interaction between frontal and apical theta oscillations and gamma activities [19]. Voegtle et al. did not find that repeated tDCS stimulation enhanced the working memory behavior of the subjects, and they discovered the impact of repeated tDCS stimulation on working memory at the neuroelectrophysiological level [20]. Meanwhile, Antonenko’s research found that tDCS did not increase working memory ability after cognitive training in patients with cognitive impairment [21]. This heterogeneity in treatment outcomes has prompted researchers to deepen their understanding of the underlying mechanisms. Therefore, the purpose of this study is to systematically explore the effect and neuroelectrophysiological mechanism of tDCS on working memory, in order to provide a clearer direction and theoretical basis for future research.

Electroencephalography (EEG) has been extensively employed in studies related to working memory due to its non-invasive characteristic, low operational cost, and high temporal resolution [22,23]. Some of the EEG bands have been found to play an important role in working memory. Cognitive neuroscientists reveal that delta oscillations contribute to a range of cognitive processes and are fundamental for stimulus information processing, attentional selection, and information integration [24]. Oscillatory activity and connectivity in the delta band reflect the efficiency of information transfer between key brain regions such as the prefrontal lobe and the hippocampus, and are essential for the maintenance and renewal of working memory [25,26]. The gamma band is considered to be closely related to memory, attention, information transmission, and integration, and constitutes the basis of complex cognitive functions such as working memory [27,28]. Gamma band activity was significantly enhanced in working memory tasks, particularly in the prefrontal and parietal regions, reflecting increased synchronicity between neuronal populations and improved information processing efficiency [29,30].

In this study, EEG data from both the resting state and working memory task state were collected from participants in the working memory group as well as the true and sham tDCS groups. Firstly, the phase–amplitude coupling (PAC), power spectral density (PSD), and brain network analysis were utilized to extract the characteristics of the delta and gamma bands in the resting and task states of working memory to explore the EEG markers of working memory. Furthermore, by comparing the task state EEG characteristics and effects after active and sham stimulation of tDCS, the impact of tDCS on working memory was explored.

## 2. Materials and Methods

### 2.1. Experiment

#### 2.1.1. Participant

Thirty-six healthy adult students (18 males and 18 females, age: 23.4 ± 2.3) were recruited from Hebei University of Technology to participate in the experiment. The participants were randomly assigned to one of the three groups: working memory group, tDCS group, and sham group, with each group comprising 12 individuals (6 males and 6 females). All participants had normal vision, no color blindness, no history of brain disease, and no contraindications to tDCS. Additionally, none had undergone experimental or medical tDCS treatment within one month prior to the study. All participants were right-handed and were instructed to abstain from psychotropic substances and dietary stimulants (e.g., coffee, tea) the day before the experiment, as well as to ensure adequate sleep the night prior. Informed consent was obtained from all participants after they were fully briefed on the study’s purpose and procedures. The study was approved by the Biomedical Ethics Committee of Hebei University of Technology.

#### 2.1.2. Experiment Design

**Figure 1:**
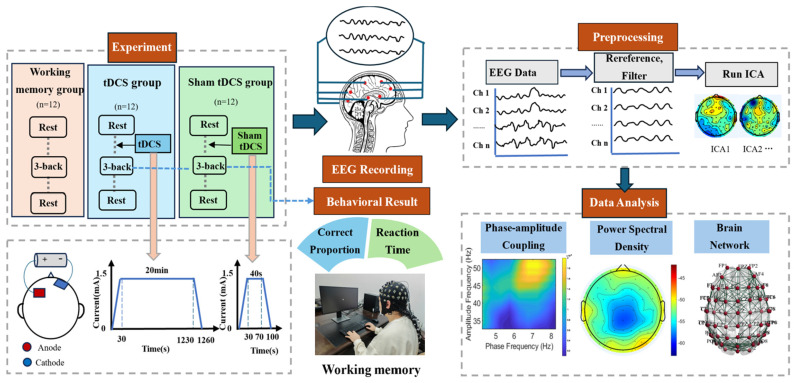
Flow diagram of the experiment.

Initially, resting-state EEG signals were recorded from all participants for 150 s prior to the 3-back working memory task. Then, participants in the tDCS group and the sham group received either true or sham tDCS stimulation targeted to the left dorsolateral prefrontal cortex (DLPFC), as per their group assignment. Subsequently, participants completed the 3-back working memory task, during which task state EEG data were collected. The working memory task consisted of four blocks, with a 120 s rest period following each block. Finally, 150 s of resting-state EEG data was acquired again after the completion of all the tasks. A flow diagram of the experiment is shown in Figure 1.

#### 2.1.3. tDCS

A transcranial direct current stimulator (DC-STIMULATOR PLUS, NeuroConn, Ilmenau, Germany) was used to stimulate the left dorsolateral prefrontal cortex (DLPFC) of the participants. The two electrodes were 5 cm × 7 cm in size and were covered with a saline-soaked sponge sleeve. Currently, the prefrontal cortex is widely recognized as one of the brain regions most closely related to working memory, especially the left dorsolateral prefrontal cortex (DLPFC) [31,32,33]. Therefore, we chose to place the anode at the location corresponding to the left dorsolateral prefrontal lobe and the cathode at the right supraorbital region. The true tDCS group received stimulation before the 3-back task, with a stimulation duration of 20 min and current intensity of 1.5 mA (fade-in/out time 30 s). The sham tDCS group had the same conditions as the true tDCS group except that the stimulus current duration was only 40 s. The participants were blinded to the tDCS condition they received.

#### 2.1.4. Three-Back Task

**Figure 2:**
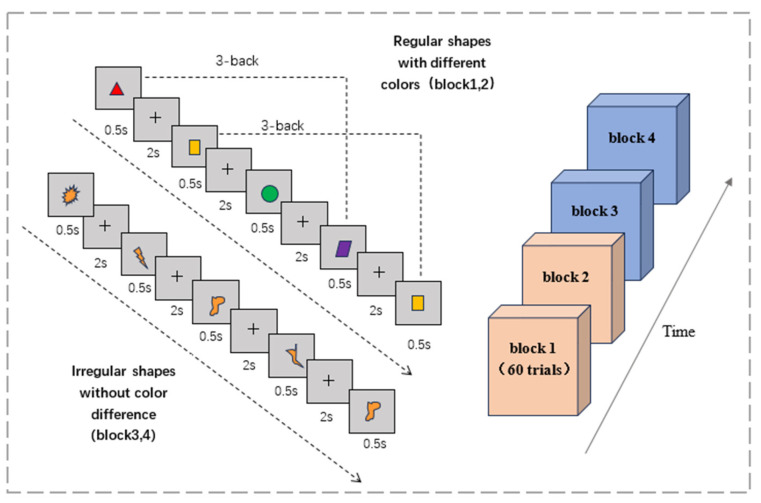
Three-back working memory task.

The three-back task consists of two types of experimental materials: regular shapes of different colors and irregular shapes of the same color. The graphic and the “+” symbol appear alternately in the center of the computer’s black background screen; the graphic appears for 0.5 s and the “+” symbol lasts for 2 s. If the current graph matched the third previous one, participants were required to press the left mouse button when the “+” symbol appeared, and press the right mouse button if the graph did not match (Figure 2). After the participants make a judgment, the next figure appears. Each block of 3-back experiments consisted of 60 trials with a 50% match or mismatch ratio. The reaction time and accuracy rate of each subject on the 3-back task were recorded.

### 2.2. Data Acquisition and Preprocessing

EEG data were recorded using an ESI-128EEG/ERP system (SynAmps 2, Curry 8, NeuroScan, Charlotte, NC, USA), and the working memory experimental tasks were performed using the E-Prime v3.0 software (Psychology Software Tools, Pittsburgh, PA, USA). The electrode impedance was kept below 5 KΩ during the experiment, and the 60-channel EEG data were recorded at a sampling rate of 1000 Hz.

Before performing EEG feature extraction, the raw data first need to be preprocessed. The data were re-referenced using M1 and M2 as reference electrodes, then filtered (0.2~80 Hz) to remove noise, and independent component analysis (ICA) was performed to remove artifacts. Next, the EEG signals were filtered to obtain signals in the delta (0.5–4 Hz) and gamma (30–50 Hz) frequency bands.

### 2.3. Analysis

In this study, delta (0.5–4 Hz) and gamma (30–50 Hz) EEG signals were analyzed and features were extracted by phase–amplitude coupling, power spectral density, and brain network analysis.

Phase–amplitude coupling (PAC) focuses on the coupling between the low-frequency phase and high-frequency amplitude of EEG, which can indicate the information exchange and neural interactions during cognitive processes [34,35]. Previous studies have found that phase–amplitude coupling between the low-frequency phase and the high-frequency amplitude is closely related to the brain’s processing, transmission, and integration of information during cognitive processes, potentially representing an underlying communication mechanism of the brain [36,37].

The modulation index (MI) was calculated to indicate the strength of delta–gamma PAC. All possible phases in the range from −180° to 180° need to be divided into bins. In this experiment, the phase was divided into 18 bins, consistent with the number of bins used by Tort [38]. The modulation index is shown as follows:

(1) $$p(j)=\frac{\bar{a}}{\sum_{k=1}^{N}\bar{a_{k}}}$$

(2) $$H(p)=-\sum_{j=1}^{N}p(j)\log p(j)$$

(3) KL(U,X)=logN−H(p)

(4) $$MI=\frac{KL(U,X)}{\log N}$$

where $\bar{a}$ is the average amplitude of a single bin, $\sum_{k=1}^{N}\bar{a_{k}}$ is the sum of the average amplitudes, *j* is label of the bin, *k* is the running index of the bin, *N* is the total number of bins, and *p* is a vector of normalized average amplitudes for each phase box.

The power spectrum analysis method is a very important spectral analysis method, which mainly explores the variation of signal energy with frequency and the distribution of signal energy in the frequency domain. Welch’s method is one of the most commonly used methods for PSD calculation. The formula is shown below:

(5) $$W(n)=(1-a)-a\times \cos(\frac{2\pi n}{N-1}),0\leq n\leq N-1$$

(6) $$X_{i}(k)=\sum_{n=0}^{M-1}x_{i}(n)W(n)e^{-jn\frac{2\pi k}{M}}$$

(7) $$P_{xx}(k)=\frac{1}{ML}\sum_{i=1}^{L}\left|X_{i}(k\right){|}^{2}$$

where *W*(*n*) is the expression of the Hamming window for a signal of length *N*, assuming that each segment of the signal *x*(*n*) is *x_i_*(*n*) after segmentation, *X_i_*(*k*) is the expression of the discrete Fourier transform (DFT) after adding the window, and the self-power spectral density of this signal is *P_xx_*(*k*). In order to facilitate comparison, the logarithm of the amplified average self-power spectral density was selected for analysis and significance comparison when feature extraction was carried out.

In this experiment, the Pearson correlation coefficient is used to construct the brain network, and a suitable threshold was chosen to binarize the correlation coefficient matrix to obtain the topology of the brain network and the related brain network coefficients. Network threshold selection was guided by some essential criteria: the absence of isolated nodes, maintenance of appropriate network density, and preservation of topological parameter validity. The definition of Pearson correlation coefficient *r* is shown below:

(8) $$r=\frac{\sum_{i=1}^{n}\left(x_{i}-\bar{x})(y_{i}-\bar{y}\right)}{\sqrt{\sum_{i=1}^{n}{\left(x_{i}-\bar{x}\right)}^{2}}\sqrt{\sum_{i=1}^{n}{\left(y_{i}-y\right)}^{2}}}$$

where $x_{i}$ and $y_{i}$ are the values of the corresponding variables of the two signals, $x_{i}$ and $y_{i}$ are the average values of the variables, and *n* is the length of the signal.

The node degree and clustering coefficient of the brain network were chosen to be analyzed in this study. Node degree refers to the number of connecting edges between a node and other nodes; the larger the degree of a node, the more important the node’s position in the network, as shown in Equation (9). The clustering coefficient is another important parameter of the metric network which indicates the level of interconnectivity between a node and its neighboring nodes and measures the degree of clustering of the network. The calculation formula is shown in Equation (10).

(9) $$k_{i}=\sum_{j=1}^{n}a_{ij}$$

(10) $$C_{i}=\frac{2E_{i}}{k_{i}(k_{i}-1)}$$

where *n* denotes the total number of network nodes, $a_{ij}$ denotes the connectivity between two nodes, and $E_{i}$ is the number of edges that are actually connected between a node and its neighboring nodes.

## 3. Results

### 3.1. EEG Feature of Working Memory Task States

#### 3.1.1. Data Analysis Based on Phase–Amplitude Coupling

In this study, the modulation index (MI) was calculated to represent the delta–gamma phase–amplitude coupling of each state. Significance tests (Wilcoxon signed-rank test, *p* < 0.05) were performed on PAC results of the resting state and task state. It was found that the MI of each node in the task state was significantly higher than that in the resting state (*p* < 0.05).

**Figure 3:**
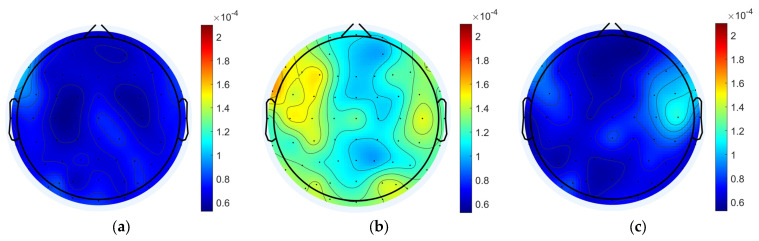
Average MI for resting and task states. (**a**) Pre-task resting state MI. (**b**) Task state MI. (**c**) Post-task resting state MI.

The MI for each node of the 12 participants was averaged, as shown in Figure 3. The whole-brain mean MI was overall significantly higher in the task state than in the pre-task resting state and the post-task resting state (pre-task resting state: 6.954 × 10^−5^ ± 8.438 × 10^−6^; task state: 1.252 × 10^−4^ ± 1.605 × 10^−5^; post-task resting state: 7.263 × 10^−5^ ± 9.337 × 10^−6^; pre-task resting state vs. task state: *p* = 1.63 × 10^−11^ < 0.001; post-task resting state vs. task state: *p* = 1.62 × 10^−11^ < 0.001; pre-task resting state vs. post-task resting state: *p* = 0.576). During the working memory task, there was a general trend toward higher coupling values in the left and right frontal regions, as well as in some of the posterior parietal and occipital regions, compared to other brain regions. When comparing the dorsolateral prefrontal lobe, where coupling was higher, the result showed that participants exhibited higher MI in the left frontal region compared to the right frontal region.

#### 3.1.2. Data Analysis Based on Power Spectral Density

**Figure 4:**
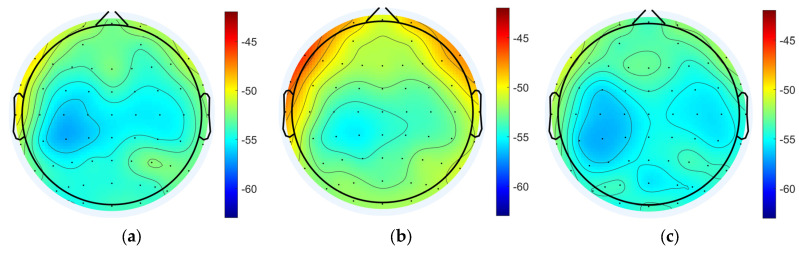

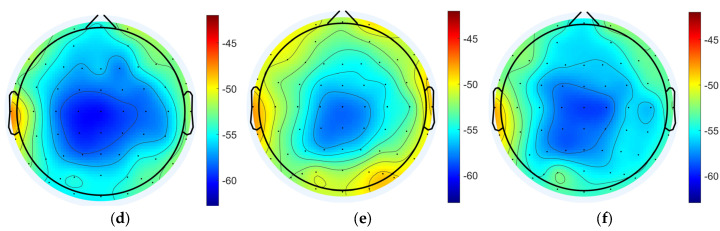
Power spectral density analysis for each state. (**a**) Pre-task resting state delta band power spectral density. (**b**) Task state delta band power spectral density. (**c**) Post-task resting state delta band power spectral density. (**d**) Pre-task resting state gamma band power spectral density. (**e**) Task state gamma band power spectral density. (**f**) Post-task resting gamma band power spectral density.

The power spectral density of EEG signals in each state was analyzed and the results showed that the power spectral densities of delta band in the task state were generally enhanced compared to the resting states, and the differences were significant (pre-task resting state: −53.755 ± 1.584 dB; task state: −51.708 ± 2.062 dB; post-task resting state: −54.315 ± 1.291 dB; pre-task resting state vs. task state: *p* = 1.31 × 10^−11^ < 0.001; post-task resting state vs. task state: *p* = 1.62 × 10^−11^ < 0.001; pre-task resting state vs. post-task resting state: *p* = 0.06 > 0.05; the above results are the whole-brain mean PSD). The power spectral density of the task state in the gamma band was also enhanced overall and was significantly different (pre-task resting state: −55.381 ± 2.662 dB; task state: −52.806 ± 2.691 dB; post-task resting state: −55.031 ± 2.205 dB; pre-task resting state vs. task state: *p* = 1.62 × 10^−11^ < 0.001; post-task resting state vs. task state: *p* = 1.63 × 10^−11^ < 0.001; pre-task resting state vs. post-task resting state: *p* = 0.23 > 0.05). No statistically significant differences in delta and gamma band PSD values were detected between pre-task and post-task resting state. Regional analysis revealed distinct spatial patterns of PSD variations across frequency bands. For delta band oscillations, the most pronounced differences were localized to bilateral dorsolateral prefrontal cortices (DLPFCs), whereas gamma band PSD alterations were predominantly observed in frontal regions and parietal and occipital areas (Figure 4).

#### 3.1.3. Data Analysis Based on Brain Network

**Figure 5:**
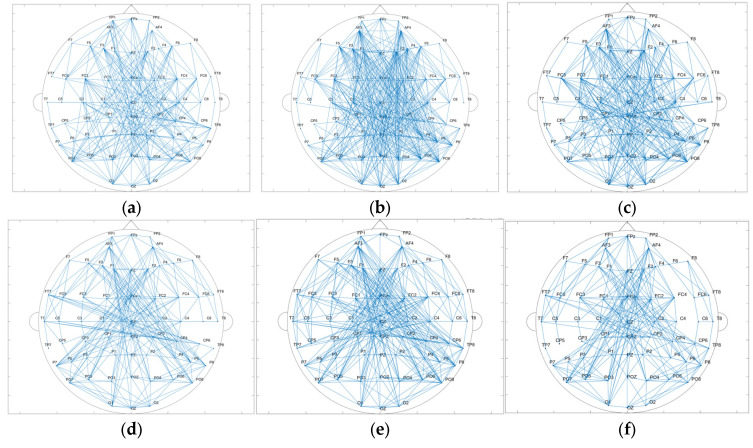
(**a**) Topological map of the gamma band in the pre-task resting state. (**b**) Topological map of the gamma band in the task state. (**c**) Topological map of the gamma band in the post-task resting state. (**d**) Topological map of the delta band in the pre-task resting state. (**e**) Topological map of the delta band in the task state. (**f**) Topological map of the delta band in the post-task resting state.

**Figure 6:**
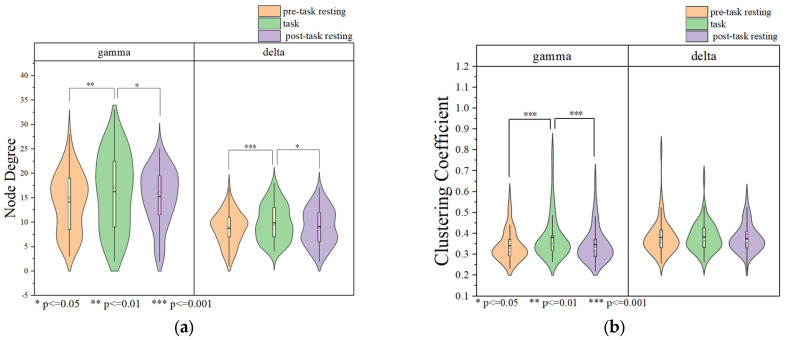
Brain network analysis for each state. (**a**) Node degree of delta and gamma bands. (**b**) Clustering coefficient of delta and gamma bands.

The results of the brain network in delta and gamma band are shown in Figure 5. Node degree and clustering coefficients were calculated based on brain networks constructed with Pearson’s correlation coefficients in delta and gamma frequency bands, and the results are shown in Figure 6. The node degrees of the working memory task state in the gamma band were significantly higher than those of the pre-task resting state and the post-task resting state (pre-task resting state: 14.100 ± 5.979; task state: 16.233 ± 5.857; post-task resting state: 15.342 ± 5.711; pre-task resting state vs. task state: *p* = 9.81 × 10^−5^ < 0.001; post-task resting state vs. task state: *p* = 0.03 < 0.05; pre-task resting state vs. post-task resting state: *p* = 0.068 > 0.05). Similarly, clustering coefficients were significantly elevated during the task state (pre-task resting state: 0.681 ± 0.146; task state: 0.771 ± 0.248; post-task resting state: 0.693 ± 0.171; pre-task resting state vs. task state: *p* = 3.71 × 10^−6^ <0.001; post-task resting state vs. task state: *p* = 6.85 × 10^−5^ < 0.001; pre-task resting state vs. post-task resting state: *p* = 0.244 > 0.05). Consistent with the gamma band findings, the delta band node degree during the working memory task showed significant increases compared to both pre-task and post-task resting states (pre-task resting state: 9.033 ± 3.369; task state: 10.325 ± 3.677; post-task resting state: 9.254 ± 3.653; pre-task resting state vs. task state: *p* = 6.48 × 10^−4^ <0.001; post-task resting state vs. task state: *p* = 0.03 < 0.05; pre-task resting state vs. post-task resting state: *p* = 0.200 > 0.05). The clustering coefficients in the delta band showed no statistically significant differences between the task state and resting state (pre-task resting state: 0.762 ± 0.205; task state: 0.767 ± 0.141; post-task resting state: 0.751 ± 0.135; pre-task resting state vs. task state: *p* = 0.418 > 0.05; post-task resting state vs. task state: *p* = 0.346 > 0.05; pre-task resting state vs. post-task resting state: *p* = 0.880 > 0.05). No statistically significant differences in node degree or clustering coefficients were observed between pre-task and post-task resting states for both delta and gamma frequency bands. The correlation thresholds were set at 0.32 for delta band networks and 0.56 for gamma band networks.

### 3.2. EEG Feature and Behavioral Result of Working Memory Task in True/Sham tDCS Group

Electroencephalographic (EEG) signals were acquired during three experimental conditions: pre-task resting state, task performance, and post-task resting state, for both active and sham tDCS groups. Comprehensive analyses employing phase–amplitude coupling, power spectral density, and brain network algorithms revealed more pronounced tDCS-induced modulations during the task state compared to resting states. Based on these findings, we focused our subsequent analysis on task-related EEG differences between active and sham tDCS groups to further elucidate the effects of tDCS on neural activity during working memory tasks.

#### 3.2.1. Three-Back Behaviorral Result

**Figure 7:**
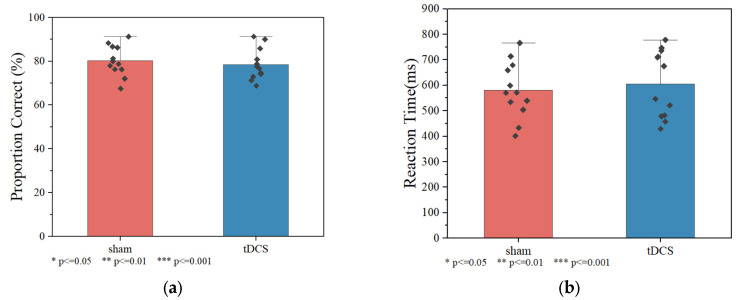
Correct proportion and reaction time on the 3-back task for each group. (**a**) Correct proportion. (**b**) Reaction time.

The Wilcoxon signed-rank test was used to evaluate the reaction time and correctness of the working memory task between the tDCS group and sham group, as shown in Figure 7 There was no significant difference in reaction time among all groups. Behavioral performance analysis revealed no significant differences between active and sham stimulation groups. Reaction times were comparable between groups (sham: 581.010 ± 89.585 ms; tDCS: 598.990 ± 93.044 ms; *p* = 0.814). Accuracy rates showed no group differences (sham: 80.210 ± 6.967%; tDCS: 79.543 ± 7.201%; *p* = 0.638).

#### 3.2.2. Data Analysis Based on Phase–Amplitude Coupling

**Figure 8:**
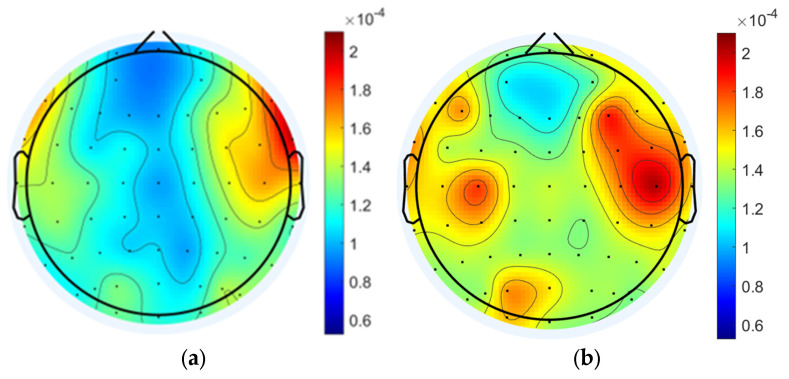

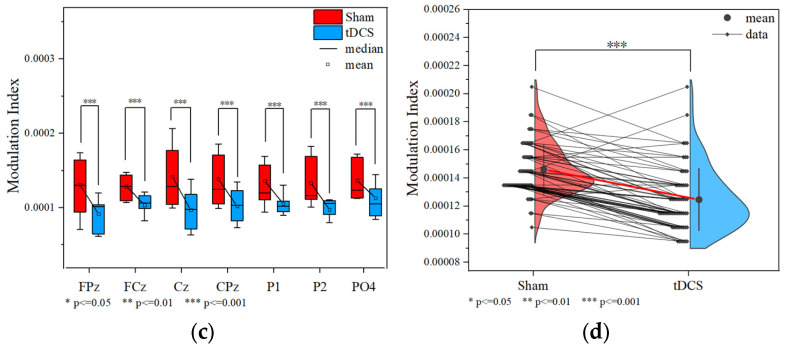
Comparison of MI in the tDCS group and the sham group. (**a**) Task state MI of the tDCS group. (**b**) Task state MI of the sham group. (**c**) Nodes with significant differences in MI between the tDCS and sham groups. (**d**) The average MI change between the sham and tDCS groups.

Comparing the MI of the 60 nodes of the tDCS group and the sham group during the working memory task, there was a tendency for MI values in the right frontal area to be slightly higher than those in the left frontal area in the tDCS group (Figure 8a,b). Wilcoxon signed-rank tests conducted on task state MI values identified significant group differences at specific nodes (FPz, FCz, Cz, CPz, P1, P2, PO4), and the sham group demonstrating consistently higher MI values than the tDCS group in all these nodes (Figure 8c). At the global level, the whole-brain mean MI during the working memory task was significantly higher in the sham group compared to the tDCS group (sham: 1.461 × 10^−4^ ± 1.785 × 10^−5^; tDCS: 1.247 × 10^−4^ ± 2.208 × 10^−5^; *p* = 3.71 × 10^−9^ < 0.001), as shown in Figure 8d. The EEG analysis revealed significantly lower mean MI values in the tDCS group compared to the sham group, suggesting that anodal tDCS may suppress delta–gamma phase–amplitude coupling. This interpretation is further supported by the observed reduction in MI values in the left frontal region relative to the right frontal region in the tDCS group.

#### 3.2.3. Data Analysis Based on Power Spectral Density

**Figure 9:**
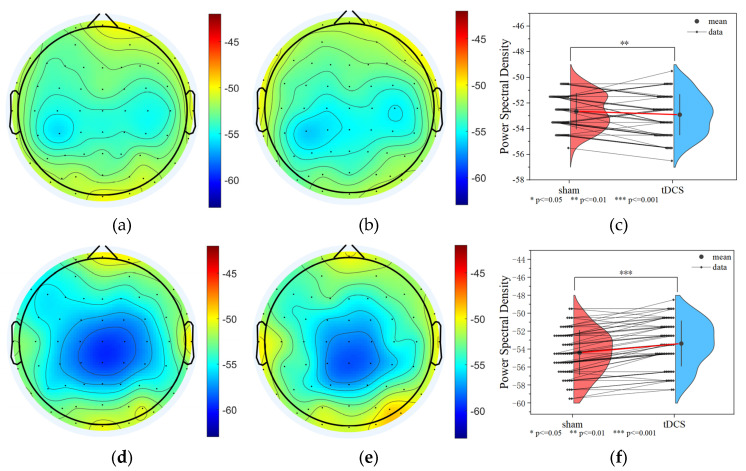
PSD results for the tDCS group and the sham group. (**a**) Delta band PSD of the sham group (**b**) Delta band PSD of the tDCS group. (**c**) The average PSD change between the sham and tDCS groups in the delta band. (**d**) Gamma band PSD of the sham group. (**e**) Gamma band PSD of the tDCS group. (**f**) The average PSD change between the sham and tDCS groups in the gamma band.

The power spectral density of the task state EEG after active and sham stimulation was analyzed, and it was found that the PSD of the tDCS group was smaller overall than that of the sham group in the delta band (sham: −52.634 ± 1.387 dB; tDCS: −53.603 ± 1.586 dB; *p* = 9.8 × 10^−3^ < 0.01), but the PSD of the tDCS group was larger overall than that of the sham group in the gamma band (sham: −54.384 ± 2.475 dB; tDCS: −53.064 ± 2.526 dB; *p* = 9.8 × 10^−3^ < 0.01; *p* = 5.974 × 10^−10^ < 0.001), as shown in Figure 9.

#### 3.2.4. Data Analysis Based on the Brain Network

**Figure 10:**
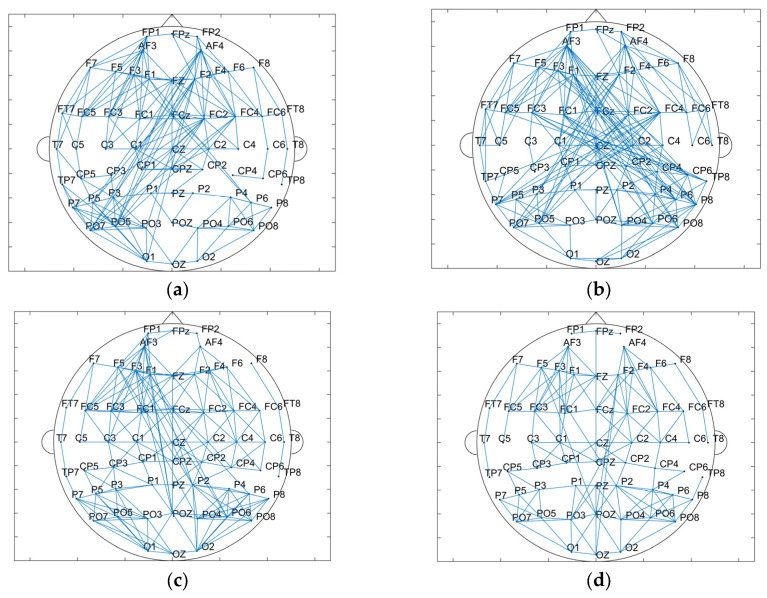

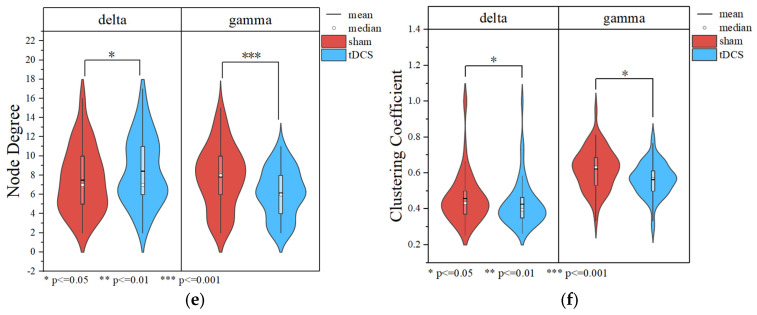
(**a**) Topological map of the delta band in the sham group. (**b**) Topological map of the delta band in the tDCS group. (**c**) Topological map of the gamma band in the sham group. (**d**) Topological map of the gamma band in the tDCS group. (**e**) Comparison of the node degree between the sham and tDCS groups. (**f**) Comparison of clustering coefficients between the sham and tDCS groups.

The results of the brain network in delta and gamma bands are shown in Figure 10. In the delta band, the core nodes with a higher node degree in the tDCS group and the sham group are mostly concentrated in the frontal region and part of the occipital and parietal regions. Additionally, it can be more intuitively found that the nodes in the frontal lobe area are more closely connected to the nodes in the parietal occipital lobe area. Comparing the nodal degrees and clustering coefficients of the tDCS group and the sham group in the delta band, the nodal degrees of the tDCS group were significantly larger (sham: 7.500 ± 3.397; tDCS: 8.433 ± 3.524; *p* = 0.02 < 0.05), the clustering coefficients of the tDCS group were lower than those of the sham group (sham: 0.458 ± 0.141; tDCS: 0.427 ± 0.126; *p* = 0.04 < 0.05), and there was a greater enhancement of connectivity between the left frontal region and the parietal and occipital regions.

Similar to the delta band, the core nodes with a higher node degree in the tDCS group and the sham group in the gamma band were likewise mostly concentrated in the frontal region and part of the parietal and occipital regions. Compared with the sham group, the tDCS group in the gamma band has an overall decrease in the node degree (sham: 7.833 ± 3.258; tDCS: 6.167 ± 2.505; *p* = 2.48 × 10^−5^ < 0.001) and an overall decrease in the clustering coefficient (sham: 0.622 ± 0.113; tDCS:0.565 ± 0.092; *p* = 0.03 < 0.05) in comparison with the sham group. The correlation thresholds were set at 0.43 for delta band networks and 0.67 for gamma band networks.

## 4. Discussion

By comparing and analyzing the EEG signals of the working memory task state and the resting state, we observed that the phase–amplitude coupling between the delta and gamma bands in the left and right frontal regions was higher during the working memory task. Phase–amplitude coupling between low and high frequencies in EEG is considered to be closely related to working memory [39,40]. Additionally, the power spectral densities of the delta and gamma bands were also more elevated compared with that of the resting state, which may indicate that the frontal regions possess more active brain activity during working memory. These phenomena were also revealed in some parietal and occipital regions. From these results, it can be inferred that the left and right frontal regions, together with the partial parietal and occipital regions, may play a more critical role in working memory. It has been shown that activation of the DLPFC is associated with working memory load, which is supported by a reentrant circuit between the DLPFC and the posterior cortex [41]. During working memory tasks, the frontal, occipital, and parietal regions are interconnected and operate in a coordinated manner, each assuming distinct functional roles. The frontal area is widely recognized as being closely related to working memory, and it performs a complex set of neural activities to store and process working memory-related content and influence working memory capacity [42]. Due to the visual nature of the task, which required participants to focus on picture material, the occipital region—an area closely associated with vision—exhibited heightened activity. Additionally, numerous studies have indicated that the parietal region plays a crucial role in integrating sensory information for higher cognitive functions and is closely linked to working memory retrieval [43]. These findings confirm that multiple brain regions coordinate to execute the higher cognitive processes involved in working memory tasks.

In the experiment, we also assessed the accuracy and the reaction time of working memory task in the tDCS and sham groups. Our research results show that no behavioral changes in working memory tasks were observed after either active or sham tDCS, but tDCS does have an impact on the neuroelectrophysiological mechanism of working memory. Research has found that in the effect of tDCS on working memory, neural activity can more sensitively perceive the effects of tDCS compared to behavioral measures [44]. While we had anticipated that we would observe correlations between neurophysiological changes and cognitive function, the absence of behavioral effects in working memory performance precludes us from establishing direct links between the observed neural changes and behavioral modifications. What can be conclusively determined is that tDCS exerts a regulatory effect on the neuroelectrophysiological mechanisms of working memory. Nevertheless, whether this neuromodulation can translate into observable cognitive enhancement remains to be verified. Future investigations should explore additional tDCS protocols (such as multiple-session tDCS and multi-site tDCS), as well as other exogenous stimulation approaches or endogenous modulation techniques (e.g., transcranial alternating current stimulation, transcranial magnetic stimulation, and neurofeedback training), to further elucidate their effects on working memory.

After tDCS stimulation, although the MI during the working memory task was significantly higher than during the resting state, consistent with the overall changes observed in the working memory group, new alterations emerged when comparing the brain regions where MI changes were more pronounced between the working memory group and the tDCS/sham groups. During the task state in the working memory group, participants showed a slightly higher MI in the left frontal region compared to the right frontal region, whereas participants in the tDCS group showed a trend toward a higher MI in the right frontal region. This may be attributed to the application of the tDCS anode to the left DLPFC. Comparing the changes in the working memory task state following true or sham tDCS stimulation revealed an overall trend of reduced MI in the tDCS group compared to the sham group. True tDCS stimulation showed a more pronounced inhibitory effect on the delta–gamma phase–amplitude coupling.

Regarding delta–gamma phase–amplitude coupling in working memory, previous studies have indicated that the delta phase modulates the amplitude of high-frequency theta and gamma oscillations through a nested mechanism [45]. Specifically, the phase of delta oscillations modulates the amplitude of theta oscillations, which in turn modulates the amplitude of gamma oscillations. Due to the complexity of the influence mechanism of tDCS on PAC, further studies are still needed. Furthermore, we found that tDCS active stimulus caused an increase in power spectral density in the gamma band and a decrease in power spectral density in the delta band. In fact, many studies have shown that anodal tDCS can depolarize neurons and increase cortical excitability [46,47,48]. However, according to the research results in recent years, anodic tDCS does not have an activating effect on neural activities in all frequency bands. The anode tDCS of the left DLPFC was found to increase gamma band oscillations, indicating greater neural activity, but reduced delta band activity and significantly decreased its average current density [49,50]. Our research results confirm the diverse effects of tDCS on different frequency bands (increasing the power spectral density in the gamma band and reducing the power spectral density in the delta band). The improvement of high-frequency power spectral density and the suppression of low-frequency power spectral density by tDCS may be related to the frequency band-specific neurophysiological mechanism. Since PAC represents the ability of low-frequency phase to regulate high-frequency amplitude, when the intensity of delta oscillation decreases, its phase modulation ability of gamma activity may be weakened, and the increase in gamma power may change its activity mode from delta phase dependence to continuous high-frequency oscillation, thus reducing the dependence on delta phase. As a result, PAC decreases. This reduction in phase–amplitude coupling may represent the differences in the influence of tDCS on different frequency bands. The specific mechanism of action remains to be further studied.

Brain network functional connectivity is an important part of research in EEG signal analysis. When the brain carries out advanced cognitive functions, it often does not only rely on the activity of a single brain region, but multiple brain regions interact with each other in a coordinated manner and share the work. Analyzing the interconnections and influences between different brain regions in advanced cognitive functions is currently a hot topic in brain science research. It has been shown that for the maintenance of working memory itself, frontal-parietal cortex regions constitute a core circuit, but how this activity reflects the critical maintenance process remains to be explored [51]. The results of brain network data analysis in this study showed that compared to the sham group, enhanced connectivity between the left frontal region and the parieto-occipital lobe was observed in the delta band of the tDCS group, and the node degree was increased but the clustering coefficient was decreased, whereas there was a decreasing trend in the node degree and clustering coefficient of the gamma band. These changes reflect the reorganization and resource redistribution of the functional network related to working memory by tDCS. In fact, the impact of transcranial direct current stimulation (tDCS) on brain functional networks exhibits profound systemic complexity. This complexity not only stems from the nonlinear interaction between application populations, stimulation paradigms, analytical methods, and neural physiological states, but is also closely related to the dynamic properties of brain networks themselves [52,53,54]. It cannot be simply defined that tDCS affects brain networks by enhancing or weakening functional connectivity. The functional connection of the brain is a very complex and important working network, and there is still a large research space for the study of brain networks.

We would like to emphasize that the current research findings are based on the EEG results of 36 participants. The number of participants and the selection of the participant group may have imposed certain limitations on the conclusions of the experiment. Additionally, other potential confounding factors, such as individual differences and the experimental environment, may further affect the estimation of the effect size. Future studies need to verify the robustness of the results in a larger sample. Nevertheless, this study provides preliminary evidence for exploring the mechanism and impact of tDCS on working memory in healthy individuals.

## 5. Conclusions

The neuroelectrophysiological mechanism and effects of the left dorsolateral prefrontal cortex (DLPFC) tDCS on working memory were investigated in this study. The tDCS-induced modulations were primarily observed in frontal, parietal, and occipital regions. These findings suggest that tDCS has the ability to regulate the neuroelectrophysiological activities of working memory in healthy individuals, although a single session of tDCS appears insufficient to translate neural effects into behavioral improvements. The results provide theoretical insights for future studies exploring the mechanisms of tDCS in working memory and optimizing stimulation protocols.

## Data Availability

The data presented in this study are available on request from the corresponding author. The data are not publicly available due to missing consent from test subjects.
